# (1*Z*,2*E*)-*N*′-{1-[2-(4-Bromo­phen­yl)hydrazin-1-yl­idene]-1-chloro­propan-2-yl­idene}thio­phene-2-carbohydrazide

**DOI:** 10.1107/S1600536812017114

**Published:** 2012-04-25

**Authors:** Hoong-Kun Fun, Suchada Chantrapromma, Hatem A. Abdel-Aziz

**Affiliations:** a X-ray Crystallography Unit, School of Physics, Universiti Sains Malaysia, 11800 USM, Penang, Malaysia; b Crystal Materials Research Unit, Department of Chemistry, Faculty of Science, Prince of Songkla University, Hat-Yai, Songkhla 90112, Thailand; c Department of Pharmaceutical Chemistry, College of Pharmacy, King Saud University, PO Box 2457, Riyadh 11451, Saudi Arabia

## Abstract

In the title compound, C_14_H_12_BrClN_4_OS, the thienyl ring is disordered over two orientations with a site-occupancy ratio of 0.853 (2):0.147 (2). The mol­ecule is roughly planar, with the dihedral angles between the thienyl and benzene rings being 6.24 (16) and 9.7 (11)° for the major and minor components, respectively. The central fragment is almost planar [r.m.s. deviation = 0.0275 (2) Å for the ten non-H atoms]. The mean plane through this middle unit makes a dihedral angle of 2.71 (7)° with the benzene ring, whereas these values are 4.46 (15) and 7.7 (11)° for the major and minor components of the thienyl ring, respectively. In the crystal, mol­ecules are linked into dimers by pairs of N—H⋯O hydrogen bonds, forming *R*_2_^2^(8) ring motifs. These dimers are arranged into sheets parallel to the *ac* plane.

## Related literature

For bond-length data, see: Allen *et al.* (1987 ▶). For hydrogen-bond motifs, see: Bernstein *et al.* (1995 ▶). For background to and the biological activity of (1*Z*,2*E*)-*N*-(ar­yl)propane­hydrazonoyl chlorides, see: Abdel-Aziz & Mekawey (2009 ▶); Abdel-Aziz *et al.* (2010 ▶). For a related structure, see: Abdel-Aziz *et al.* (2012 ▶). For the stability of the temperature controller, see: Cosier & Glazer (1986 ▶).
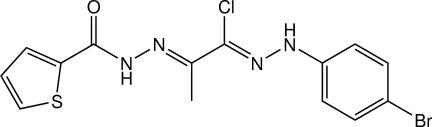


## Experimental

### 

#### Crystal data

C_14_H_12_BrClN_4_OS
*M* *_r_* = 399.79
Monoclinic, 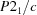

*a* = 13.5502 (14) Å
*b* = 3.8932 (4) Å
*c* = 29.816 (3) Å
β = 103.075 (2)°
*V* = 1532.1 (3) Å^3^
*Z* = 4
Mo *K*α radiation
μ = 3.00 mm^−1^
*T* = 100 K
0.38 × 0.10 × 0.06 mm

#### Data collection

Bruker APEX Duo CCD area detector diffractometer
Absorption correction: multi-scan (*SADABS*; Bruker, 2009 ▶) *T*_min_ = 0.399, *T*_max_ = 0.848
14314 measured reflections
4458 independent reflections
3378 reflections with *I* > 2σ(*I*)’
*R*_int_ = 0.037

#### Refinement

*R*[*F* ^2^ > 2σ(*F* ^2^)] = 0.031
*wR*(*F* ^2^) = 0.075
*S* = 1.05
4458 reflections
223 parameters
6 restraints
H atoms treated by a mixture of independent and constrained refinement
Δρ_max_ = 0.54 e Å^−3^
Δρ_min_ = −0.36 e Å^−3^

### 

Data collection: *APEX2* (Bruker, 2009 ▶); cell refinement: *SAINT* (Bruker, 2009 ▶); data reduction: *SAINT*; program(s) used to solve structure: *SHELXTL* (Sheldrick, 2008 ▶); program(s) used to refine structure: *SHELXTL*; molecular graphics: *SHELXTL*; software used to prepare material for publication: *SHELXTL* and *PLATON* (Spek, 2009 ▶).

## Supplementary Material

Crystal structure: contains datablock(s) global, I. DOI: 10.1107/S1600536812017114/bq2351sup1.cif

Structure factors: contains datablock(s) I. DOI: 10.1107/S1600536812017114/bq2351Isup2.hkl

Supplementary material file. DOI: 10.1107/S1600536812017114/bq2351Isup3.cml

Additional supplementary materials:  crystallographic information; 3D view; checkCIF report

## Figures and Tables

**Table 1:** Hydrogen-bond geometry (Å, °)

*D*—H⋯*A*	*D*—H	H⋯*A*	*D*⋯*A*	*D*—H⋯*A*
N4—H1*N*4⋯O1^i^	0.92 (3)	2.03 (3)	2.938 (2)	168 (2)
N1—H1*N*1⋯Cl1	0.92 (2)	2.51 (2)	2.9246 (19)	107.7 (15)

Symmetry code: (i) 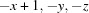.

**Fig. 1.**
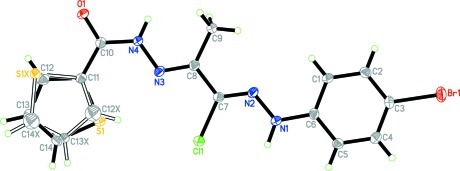
The molecular structure of the title compound with 50% probability displacement ellipsoids and the atom-numbering scheme. Open bonds show the minor component.

**Fig. 2.**
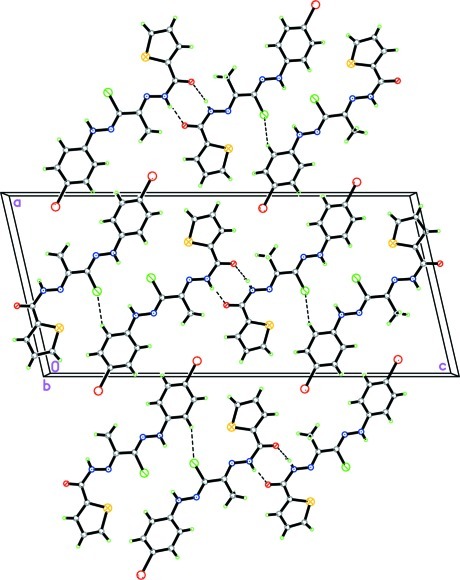
The crystal packing of the title compound viewed along the b axis. Hydrogen bonds were shown as dash lines.

## supplementary crystallographic information

### Comment

The synthesis of a new class of (1*Z*,2*E*)-*N*-(aryl)propanehydrazonoyl chlorides as analogs of the title compound have recently been reported. These derivatives were used as an economical and versatile synthetic approach for stereoselective synthesis of novel amidrazone derivatives possessing significant antifungal and antiviral potencies (AbdelAziz & Mekawey, 2009; Abdel-Aziz *et al.*, 2010). As part of our ongoing research on the bioactivity of bis-hydrazones, the title compound (I) was synthesized for a comparable study of another analog (Abdel-Aziz *et al.*, 2012). Herein we report the synthesis and crystal structure of the title compound.

In the molecule of (I) (Fig. 1), C_14_H_9_BrClN_4_OS, the thienyl ring is disordered over two positions with the refined site-occupancy ratio of 0.853 (2): 0.147 (2). The molecule is essentially planar with the dihedral angles between the thienyl and benzene rings being 6.24 (16) and 9.7 (11)° for the major and minor components, rsepectively. The middle fragment is planar with an *r.m.s*. 0.0275 (2) Å for the ten non-H atoms (C7–C10/N1–N4/O1/Cl1). The mean plane through this middle bridge makes the dihedral angle of 2.71 (7) ° with the benzene ring whereas these values are 4.46 (15) and 7.7 (11)° for the major and minor components of the thienyl ring, respectively. An intramolecular N—H···Cl hydrogen bond (Table 1) generates an S(5) ring motif (Bernstein *et al.*, 1995). The bond distances agree with the literature values (Allen *et al.*, 1987) and comparable to the related structure (Abdel-Aziz *et al.*, 2012).

In the crystal packing (Fig. 2), the molecules are linked into dimers by pairs of N—H···O hydrogen bonds forming the R^2^_2_(8) ring motifs (Bernstein *et al.*, 1995) and these dimers arranged into sheets parallel to the *ac* plane.

### Experimental

A mixture of thiophene-2-carbohydrazide (1.42 g, 10 mmol) and (*Z*)-*N*'-(4-bromophenyl)-2-oxopropanehydrazonoyl chloride (2.76 g, 10 mmol) in absolute ethanol (50 ml) was refluxed for 6 h. The reaction was then left to cool at room temperature. The solid formed was filtered off, washed with ethanol and recrystallized twice from EtOH to afford yellow needle-shaped title compound.

### Refinement

Amide H atom was located in Fourier difference maps and refined isotrpically. The remainning H atoms were positioned geometrically and allowed to ride on their parent atoms, with d(C-H) = 0.93 Å for aromatic and 0.96 Å for CH_3_ atoms. The *U*_iso_ values were constrained to be 1.5*U*_eq_ of the carrier atom for methyl H atoms and 1.2*U*_eq_ for the remaining H atoms. A rotating group model was used for the methyl groups. The thiophene ring is disordered over two sites in a 0.853 (2): 0.147 (2) occupancy ratio. Similarity restraint were used for the disordered thienyl ring. The thermal ellipsoids of each of the two pairs of atoms [C12X and C14X as well as S1X and C13X] were restrained to be the same.

### Crystal data

**Table:**

C_14_H_12_BrClN_4_OS	*F*(000) = 800
*M**_r_* = 399.79	*D*_x_ = 1.733 Mg m^−^^3^
Monoclinic, *P*2_1_/*c*	Mo *K*α radiation, λ = 0.71073 Å
Hall symbol: -P 2ybc	Cell parameters from 4458 reflections
*a* = 13.5502 (14) Å	θ = 1.4–30.0°
*b* = 3.8932 (4) Å	µ = 3.00 mm^−^^1^
*c* = 29.816 (3) Å	*T* = 100 K
β = 103.075 (2)°	Needle, yellow
*V* = 1532.1 (3) Å^3^	0.38 × 0.10 × 0.06 mm
*Z* = 4	

### Data collection

**Table:**

Bruker APEX Duo CCD area detector diffractometer	4458 independent reflections
Radiation source: sealed tube	3378 reflections with *I* > 2σ(*I*)'
Graphite monochromator	*R*_int_ = 0.037
φ and ω scans	θ_max_ = 30.0°, θ_min_ = 1.4°
Absorption correction: multi-scan (*SADABS*; Bruker, 2009)	*h* = −19→19
*T*_min_ = 0.399, *T*_max_ = 0.848	*k* = −2→5
14314 measured reflections	*l* = −41→41

### Refinement

**Table:**

Refinement on *F*^2^	Primary atom site location: structure-invariant direct methods
Least-squares matrix: full	Secondary atom site location: difference Fourier map
*R*[*F*^2^ > 2σ(*F*^2^)] = 0.031	Hydrogen site location: inferred from neighbouring sites
*wR*(*F*^2^) = 0.075	H atoms treated by a mixture of independent and constrained refinement
*S* = 1.05	*w* = 1/[σ^2^(*F*_o_^2^) + (0.0313*P*)^2^ + 0.3052*P*] where *P* = (*F*_o_^2^ + 2*F*_c_^2^)/3
4458 reflections	(Δ/σ)_max_ = 0.001
223 parameters	Δρ_max_ = 0.54 e Å^−^^3^
6 restraints	Δρ_min_ = −0.36 e Å^−^^3^

### Special details

**Table:**

Experimental. The crystal was placed in the cold stream of an Oxford Cryosystems Cobra open-flow nitrogen cryostat (Cosier & Glazer, 1986) operating at 100.0 (1) K.
Geometry. All esds (except the esd in the dihedral angle between two l.s. planes) are estimated using the full covariance matrix. The cell esds are taken into account individually in the estimation of esds in distances, angles and torsion angles; correlations between esds in cell parameters are only used when they are defined by crystal symmetry. An approximate (isotropic) treatment of cell esds is used for estimating esds involving l.s. planes.
Refinement. Refinement of F^2^ against ALL reflections. The weighted R-factor wR and goodness of fit S are based on F^2^, conventional R-factors R are based on F, with F set to zero for negative F^2^. The threshold expression of F^2^ > 2sigma(F^2^) is used only for calculating R-factors(gt) etc. and is not relevant to the choice of reflections for refinement. R-factors based on F^2^ are statistically about twice as large as those based on F, and R- factors based on ALL data will be even larger.

### Fractional atomic coordinates and isotropic or equivalent isotropic displacement parameters (Å^2^)

**Table:**

	*x*	*y*	*z*	*U*_iso_*/*U*_eq_	Occ. (<1)
Cl1	0.46435 (3)	0.69142 (13)	0.184855 (16)	0.01888 (11)	
Br1	1.077635 (15)	0.50439 (6)	0.376036 (7)	0.02669 (7)	
O1	0.38556 (10)	0.1793 (4)	−0.02052 (4)	0.0194 (3)	
N1	0.66736 (13)	0.5947 (5)	0.24295 (6)	0.0197 (4)	
N2	0.65558 (12)	0.4813 (4)	0.19957 (5)	0.0174 (3)	
N3	0.47407 (12)	0.3945 (4)	0.09546 (5)	0.0158 (3)	
N4	0.46842 (12)	0.2636 (5)	0.05229 (5)	0.0162 (3)	
C1	0.84509 (14)	0.4225 (5)	0.25890 (7)	0.0176 (4)	
H1A	0.8369	0.3350	0.2293	0.021*	
C2	0.93831 (15)	0.4030 (5)	0.28961 (7)	0.0180 (4)	
H2A	0.9929	0.3003	0.2808	0.022*	
C3	0.95018 (14)	0.5373 (5)	0.33370 (7)	0.0171 (4)	
C4	0.86974 (14)	0.6913 (5)	0.34736 (7)	0.0182 (4)	
H4A	0.8786	0.7839	0.3768	0.022*	
C5	0.77589 (14)	0.7069 (5)	0.31702 (7)	0.0174 (4)	
H5A	0.7212	0.8057	0.3263	0.021*	
C6	0.76335 (14)	0.5748 (5)	0.27276 (7)	0.0158 (4)	
C7	0.57143 (14)	0.5059 (5)	0.17035 (6)	0.0165 (4)	
C8	0.56220 (14)	0.3761 (5)	0.12343 (6)	0.0160 (4)	
C9	0.65679 (14)	0.2370 (6)	0.11173 (7)	0.0204 (4)	
H9A	0.6666	0.3445	0.0841	0.031*	
H9B	0.6502	−0.0067	0.1071	0.031*	
H9C	0.7139	0.2844	0.1365	0.031*	
C10	0.38400 (13)	0.2968 (5)	0.01792 (6)	0.0151 (4)	
C11	0.29185 (14)	0.4686 (5)	0.02516 (6)	0.0149 (4)	
S1	0.26973 (5)	0.6374 (2)	0.07517 (3)	0.01674 (17)	0.854 (2)
C12	0.2069 (3)	0.5033 (13)	−0.01037 (15)	0.0204 (8)	0.854 (2)
H12A	0.2047	0.4293	−0.0402	0.024*	0.854 (2)
C13	0.1241 (3)	0.6605 (15)	0.00274 (13)	0.0175 (7)	0.854 (2)
H13B	0.0616	0.7008	−0.0171	0.021*	0.854 (2)
C14	0.1476 (2)	0.7459 (12)	0.04819 (11)	0.0174 (7)	0.854 (2)
H14B	0.1024	0.8517	0.0631	0.021*	0.854 (2)
S1X	0.2004 (5)	0.498 (2)	−0.02227 (19)	0.0144 (13)*	0.146 (2)
C12X	0.2653 (17)	0.592 (8)	0.0633 (8)	0.039 (8)*	0.146 (2)
H12B	0.3082	0.5916	0.0924	0.047*	0.146 (2)
C13X	0.1671 (17)	0.721 (8)	0.0541 (8)	0.0144 (13)*	0.15
H13A	0.1352	0.8097	0.0760	0.017*	0.146 (2)
C14X	0.125 (2)	0.698 (13)	0.0090 (9)	0.039 (8)*	0.15
H14A	0.0607	0.7818	−0.0042	0.047*	0.146 (2)
H1N4	0.5211 (19)	0.138 (7)	0.0459 (8)	0.035 (7)*	
H1N1	0.6170 (15)	0.722 (6)	0.2508 (7)	0.013 (5)*	

### Atomic displacement parameters (Å^2^)

**Table:**

	*U*^11^	*U*^22^	*U*^33^	*U*^12^	*U*^13^	*U*^23^
Cl1	0.0152 (2)	0.0249 (3)	0.0170 (2)	0.00090 (19)	0.00470 (17)	−0.0032 (2)
Br1	0.01893 (10)	0.03023 (13)	0.02601 (12)	0.00166 (9)	−0.00517 (8)	−0.00167 (10)
O1	0.0184 (6)	0.0270 (8)	0.0133 (7)	0.0001 (6)	0.0047 (5)	−0.0034 (6)
N1	0.0163 (8)	0.0294 (10)	0.0135 (8)	0.0020 (7)	0.0039 (6)	−0.0054 (7)
N2	0.0177 (8)	0.0221 (9)	0.0122 (7)	−0.0014 (7)	0.0033 (6)	−0.0019 (7)
N3	0.0172 (8)	0.0185 (8)	0.0119 (7)	−0.0016 (6)	0.0039 (6)	−0.0006 (7)
N4	0.0139 (7)	0.0230 (9)	0.0116 (7)	0.0012 (7)	0.0028 (6)	−0.0022 (7)
C1	0.0182 (9)	0.0219 (10)	0.0137 (9)	−0.0002 (7)	0.0060 (7)	−0.0019 (8)
C2	0.0159 (9)	0.0188 (10)	0.0200 (10)	−0.0006 (7)	0.0056 (8)	−0.0007 (8)
C3	0.0145 (8)	0.0174 (10)	0.0176 (9)	−0.0019 (7)	−0.0003 (7)	0.0021 (8)
C4	0.0220 (9)	0.0182 (10)	0.0138 (9)	−0.0004 (8)	0.0030 (7)	−0.0003 (8)
C5	0.0185 (9)	0.0195 (10)	0.0147 (9)	0.0016 (8)	0.0051 (7)	0.0003 (8)
C6	0.0150 (8)	0.0181 (10)	0.0142 (9)	−0.0011 (7)	0.0029 (7)	0.0011 (8)
C7	0.0150 (8)	0.0212 (10)	0.0146 (9)	−0.0018 (8)	0.0057 (7)	−0.0002 (8)
C8	0.0162 (9)	0.0182 (9)	0.0137 (9)	−0.0009 (7)	0.0039 (7)	0.0002 (8)
C9	0.0153 (8)	0.0298 (12)	0.0156 (9)	0.0026 (8)	0.0026 (7)	−0.0037 (9)
C10	0.0146 (8)	0.0174 (9)	0.0140 (9)	−0.0031 (7)	0.0043 (7)	0.0019 (8)
C11	0.0150 (8)	0.0169 (9)	0.0138 (9)	−0.0025 (7)	0.0051 (7)	0.0005 (8)
S1	0.0170 (3)	0.0194 (3)	0.0146 (4)	0.0011 (2)	0.0052 (2)	−0.0011 (3)
C12	0.0187 (14)	0.0260 (16)	0.0185 (19)	−0.0034 (11)	0.0082 (15)	−0.0041 (19)
C13	0.0126 (12)	0.0226 (19)	0.0167 (14)	−0.0015 (10)	0.0020 (9)	0.0031 (14)
C14	0.0143 (16)	0.0217 (15)	0.0170 (15)	0.0056 (13)	0.0048 (12)	0.0032 (12)

### Geometric parameters (Å, º)

**Table:**

Cl1—C7	1.7601 (19)	C8—C9	1.503 (3)
Br1—C3	1.8990 (19)	C9—H9A	0.9600
O1—C10	1.239 (2)	C9—H9B	0.9600
N1—N2	1.342 (2)	C9—H9C	0.9600
N1—C6	1.402 (2)	C10—C11	1.475 (3)
N1—H1N1	0.92 (2)	C11—C12X	1.36 (2)
N2—C7	1.272 (2)	C11—C12	1.383 (5)
N3—C8	1.295 (2)	C11—S1X	1.660 (6)
N3—N4	1.370 (2)	C11—S1	1.717 (2)
N4—C10	1.358 (2)	S1—C14	1.721 (2)
N4—H1N4	0.92 (3)	C12—C13	1.409 (5)
C1—C2	1.384 (3)	C12—H12A	0.9300
C1—C6	1.399 (3)	C13—C14	1.361 (4)
C1—H1A	0.9300	C13—H13B	0.9300
C2—C3	1.390 (3)	C14—H14B	0.9300
C2—H2A	0.9300	S1X—C14X	1.717 (19)
C3—C4	1.384 (3)	C12X—C13X	1.389 (18)
C4—C5	1.385 (3)	C12X—H12B	0.9300
C4—H4A	0.9300	C13X—C14X	1.339 (17)
C5—C6	1.391 (3)	C13X—H13A	0.9300
C5—H5A	0.9300	C14X—H14A	0.9300
C7—C8	1.466 (3)		
			
N2—N1—C6	118.94 (16)	C8—C9—H9C	109.5
N2—N1—H1N1	119.5 (13)	H9A—C9—H9C	109.5
C6—N1—H1N1	120.4 (13)	H9B—C9—H9C	109.5
C7—N2—N1	121.87 (17)	O1—C10—N4	118.37 (17)
C8—N3—N4	115.57 (16)	O1—C10—C11	119.68 (17)
C10—N4—N3	122.12 (16)	N4—C10—C11	121.95 (17)
C10—N4—H1N4	117.1 (16)	C12X—C11—C12	105.9 (9)
N3—N4—H1N4	120.7 (16)	C12X—C11—C10	132.7 (9)
C2—C1—C6	119.53 (18)	C12—C11—C10	121.2 (2)
C2—C1—H1A	120.2	C12X—C11—S1X	113.6 (9)
C6—C1—H1A	120.2	C10—C11—S1X	113.6 (3)
C1—C2—C3	119.87 (18)	C12—C11—S1	110.5 (2)
C1—C2—H2A	120.1	C10—C11—S1	128.37 (14)
C3—C2—H2A	120.1	S1X—C11—S1	118.0 (3)
C4—C3—C2	120.75 (18)	C11—S1—C14	91.51 (15)
C4—C3—Br1	119.47 (15)	C11—C12—C13	114.0 (3)
C2—C3—Br1	119.78 (14)	C11—C12—H12A	123.0
C3—C4—C5	119.64 (18)	C13—C12—H12A	123.0
C3—C4—H4A	120.2	C14—C13—C12	111.2 (3)
C5—C4—H4A	120.2	C14—C13—H13B	124.4
C4—C5—C6	120.05 (17)	C12—C13—H13B	124.4
C4—C5—H5A	120.0	C13—C14—S1	112.9 (3)
C6—C5—H5A	120.0	C13—C14—H14B	123.6
C5—C6—C1	120.15 (18)	S1—C14—H14B	123.6
C5—C6—N1	118.55 (17)	C11—S1X—C14X	89.5 (9)
C1—C6—N1	121.30 (17)	C11—C12X—C13X	112.7 (16)
N2—C7—C8	119.82 (17)	C11—C12X—H12B	123.6
N2—C7—Cl1	121.57 (15)	C13X—C12X—H12B	123.6
C8—C7—Cl1	118.61 (14)	C14X—C13X—C12X	110 (2)
N3—C8—C7	117.55 (17)	C14X—C13X—H13A	124.9
N3—C8—C9	125.54 (17)	C12X—C13X—H13A	124.9
C7—C8—C9	116.89 (16)	C13X—C14X—S1X	113.8 (19)
C8—C9—H9A	109.5	C13X—C14X—H14A	123.1
C8—C9—H9B	109.5	S1X—C14X—H14A	123.1
H9A—C9—H9B	109.5		
			
C6—N1—N2—C7	−176.96 (19)	N4—C10—C11—C12	179.3 (3)
C8—N3—N4—C10	−173.07 (18)	O1—C10—C11—S1X	−2.8 (4)
C6—C1—C2—C3	−0.6 (3)	N4—C10—C11—S1X	176.9 (3)
C1—C2—C3—C4	−0.1 (3)	O1—C10—C11—S1	177.55 (15)
C1—C2—C3—Br1	179.12 (15)	N4—C10—C11—S1	−2.7 (3)
C2—C3—C4—C5	1.1 (3)	C12X—C11—S1—C14	−32 (13)
Br1—C3—C4—C5	−178.13 (15)	C12—C11—S1—C14	0.7 (3)
C3—C4—C5—C6	−1.4 (3)	C10—C11—S1—C14	−177.4 (2)
C4—C5—C6—C1	0.7 (3)	S1X—C11—S1—C14	3.0 (4)
C4—C5—C6—N1	179.99 (19)	C12X—C11—C12—C13	2.2 (14)
C2—C1—C6—C5	0.3 (3)	C10—C11—C12—C13	177.5 (4)
C2—C1—C6—N1	−178.97 (19)	S1X—C11—C12—C13	−166 (4)
N2—N1—C6—C5	177.19 (18)	S1—C11—C12—C13	−0.8 (5)
N2—N1—C6—C1	−3.5 (3)	C11—C12—C13—C14	0.4 (7)
N1—N2—C7—C8	−179.62 (18)	C12—C13—C14—S1	0.1 (6)
N1—N2—C7—Cl1	0.1 (3)	C11—S1—C14—C13	−0.5 (4)
N4—N3—C8—C7	−178.66 (17)	C12X—C11—S1X—C14X	2 (2)
N4—N3—C8—C9	2.3 (3)	C12—C11—S1X—C14X	15 (4)
N2—C7—C8—N3	177.33 (19)	C10—C11—S1X—C14X	179.2 (19)
Cl1—C7—C8—N3	−2.4 (3)	S1—C11—S1X—C14X	−1.1 (19)
N2—C7—C8—C9	−3.5 (3)	C12—C11—C12X—C13X	−2 (3)
Cl1—C7—C8—C9	176.79 (15)	C10—C11—C12X—C13X	−176.9 (17)
N3—N4—C10—O1	178.17 (17)	S1X—C11—C12X—C13X	−1 (3)
N3—N4—C10—C11	−1.6 (3)	S1—C11—C12X—C13X	146 (15)
O1—C10—C11—C12X	173.4 (18)	C11—C12X—C13X—C14X	−2 (4)
N4—C10—C11—C12X	−6.8 (18)	C12X—C13X—C14X—S1X	4 (5)
O1—C10—C11—C12	−0.4 (4)	C11—S1X—C14X—C13X	−3 (4)

### Hydrogen-bond geometry (Å, º)

**Table:**

*D*—H···*A*	*D*—H	H···*A*	*D*···*A*	*D*—H···*A*
N4—H1*N*4···O1^i^	0.92 (3)	2.03 (3)	2.938 (2)	168 (2)
N1—H1*N*1···Cl1	0.92 (2)	2.51 (2)	2.9246 (19)	107.7 (15)

Symmetry code: (i) −*x*+1, −*y*, −*z*.
